# Lack of temporal correlations between COVID pandemic waves and the occurrence of malignant ventricular arrhythmias

**DOI:** 10.1093/europace/euad357

**Published:** 2023-12-05

**Authors:** Deborah Foltran, Céline Guilbeau-Frugier, Nathan Marimpouy, Maxime Beneyto, Miloud Cherbi, Vanina Bongard, Philippe Maury

**Affiliations:** Cardiology, University Hospital Toulouse, 1, avenue du Pr Jean Poulhès - TSA 50032- 31059 Toulouse cedex 9, France; Cardiology, University Hospital Toulouse, 1, avenue du Pr Jean Poulhès - TSA 50032- 31059 Toulouse cedex 9, France; Cardiology, University Hospital Toulouse, 1, avenue du Pr Jean Poulhès - TSA 50032- 31059 Toulouse cedex 9, France; Cardiology, University Hospital Toulouse, 1, avenue du Pr Jean Poulhès - TSA 50032- 31059 Toulouse cedex 9, France; Cardiology, University Hospital Toulouse, 1, avenue du Pr Jean Poulhès - TSA 50032- 31059 Toulouse cedex 9, France; Cardiology, University Hospital Toulouse, 1, avenue du Pr Jean Poulhès - TSA 50032- 31059 Toulouse cedex 9, France; Cardiology, University Hospital Toulouse, 1, avenue du Pr Jean Poulhès - TSA 50032- 31059 Toulouse cedex 9, France

**Keywords:** COVID, Ventricular arrhythmias, Pandemic, Sudden death

## Abstract

**Aims:**

Correlations between malignant ventricular arrhythmias and the COVID waves have never been investigated.

**Methods and results:**

Prevalence of malignant ventricular arrhythmias/sudden cardiac death has been correlated to the four COVID waves between the onset of pandemic and end 2021. No significant correlation was present in the temporal evolution of both COVID patients/positive tests and incidence of malignant ventricular arrhythmias, which tended to decrease after vaccination onset.

**Conclusion:**

We present evidence of complex higher-order periodicities and the co-existence of such regions with stable non-chaotic areas in *ex vivo* human hearts. We infer that the oscillation of the calcium cycling machinery is the primary mechanism of higher-order dynamics. These higher-order regions may act as niduses of instability and may provide targets for substrate-based ablation of VF.

Association between COVID-19 and sudden death (SD) has been repetitively underlined at the very onset of the pandemic. However, causal relationship between SD and COVID-19 remained sometimes elusive, whereas evolution of the incidence of malignant ventricular arrhythmias (VA) regarding the succession of pandemic waves has never been investigated.

Number of hospitalizations for COVID and number of COVID positive tests (pharyngeal swab) for the whole Midi-Pyrénées area (retrieved from the Agence Regionale de Santé) were used as a surrogate of regional pandemic and grouped according to the four successive pandemic waves observed in this area between March 2020 and end 2021.

Number of electrical storm (ES), ablations for ventricular tachycardia (VT) (outside ES), and number of cardiac SD at the Toulouse University Hospital were grouped according to the same four pandemic waves. Cardiac SD was defined at necropsy by the lack of any extra cardiac cause in patients deceased from SD. Since pandemic waves were not equal in duration, the number of COVID patients, COVID positive tests, ES, SD, and VT ablations for each wave were then divided by the duration of the considered wave and expressed per month. Monthly number of STEMI referred at our institution was also collected. Monthly number of COVID-vaccinations for the whole area was also available since onset of vaccination in 2021. COVID and vaccination status were available for each living patient.

Temporal evolution of all these parameters was then compared. Relationships between two time series were evaluated by the cross-correlation function (R Studio®) and correlation coefficients were assessed by multiple correlation testing (Excell tool pack®). No approval for ethical committee was sought (epidemiological anonymous data).

There was no significant correlation between the temporal evolutions of hospitalizations for COVID or COVID positive tests and the number of ES, VT ablations, cardiac SD, or total VA according to the four COVID waves experienced in our area (*P* = ns for each, *r* < 0.7). Lack of significant association between COVID patients or tests and STEMI was also noted. A decrease in VA seems to occur concomitantly to the vaccination (see *Figure 1*). Only four hospitalized patients for VT ablation or ES were diagnosed with COVID infection (two with ongoing, two with remote infections), and 45% of them were vaccinated after onset 2021.

**Figure 1:**
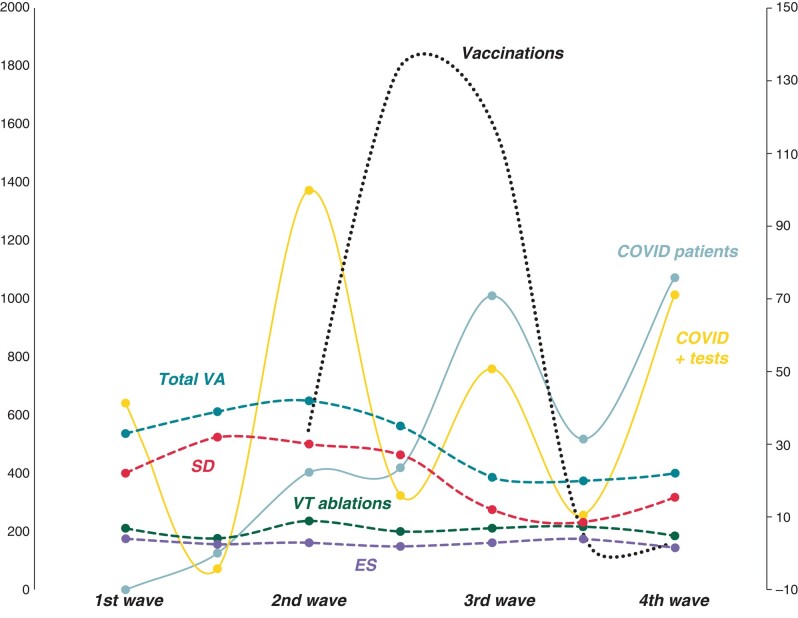
Temporal evolution of monthly number of COVID patients/tests, vaccinations, and malignant ventricular arrhythmias grouped according to the four main COVID pandemic waves. The number of vaccinated subjects or COVID tests were divided by 20 and 100 for graphical grounds. SD, sudden death; ES, electrical storm; VT, ventricular tachycardia; total VA, total ventricular arrhythmias.

Our data tends to demonstrate that COVID pandemic waves were not temporally correlated to cardiac SD and VA in patients free from COVID infection, while VA incidence rather decreased after the onset of vaccination. To the best of our knowledge, there is no previous report of temporal relationship between COVID and VA. Most previous studies had shown some relationship between COVID pandemic and out of hospital cardiac arrest (CA), but all these were performed at the very onset of pandemic and no longer association all along the course of the pandemic had been reported to date. Second, most did not either investigate the precise aetiology of SD (which could have been related to respiratory causes in a relevant part), while causal relationship with COVID infection was either not investigated or considered minor. Significant increase in CA and VA in early pandemic was noted in initial reports and significantly correlated to the incidence of COVID in the general population^1–3^ with only a limited number with proved or suspected COVID infection.^1,2,4^ In opposition, a significant 32% reduction in VA in ICD patients was noted in a large US population during the initial pandemic, potentially explained by the lockdown and its multiple and sometimes opposite consequences^5^ together with substantial reductions in the number of cardiac interventions undertaken.^6^ No further association over the following COVID waves has been reported however. Hydroxychloroquine and azithromycin, drugs sometimes used in some countries at the onset of the pandemic, have little impact on QT duration and do not induce any substrate prone to arrhythmia in COVID-19 patients with normal cardiac repolarization reserve^7^ and were not used anyway in our area.

## Conclusion

COVID pandemic waves were not related to the incidence of VA in the absence of acute or remote COVID infection. This means that the multiple consequences of social distancing (i.e. delayed medical management) did not have led to an increase in the occurrence of VA. Finally, there was no increase in VA after vaccination.

## Data Availability

Data available on demand.

## References

[1] Baldi E, Sechi GM, Mare C, Canevari F, Brancaglione A, Primi R et al COVID-19 kills at home: the close relationship between the epidemic and the increase of out-of-hospital cardiac arrests. Eur Heart J 2020;41:3045–54. 32562486 10.1093/eurheartj/ehaa508 PMC7337787

[2] Marijon E, Karam N, Jost D, Perrot D, Frattini B, Derkenne C et al Out-of-hospital cardiac arrest during the COVID-19 pandemic in Paris, France: a population-based, observational study. Lancet Public Health 2020;5:e437–43. 32473113 10.1016/S2468-2667(20)30117-1 PMC7255168

[3] Aung S, Vittinghoff E, Nah G, Lin A, Joyce S, Mann NC et al Emergency activations for chest pain and ventricular arrhythmias related to regional COVID-19 across the US. Sci Rep 2021;11:23959. 34907226 10.1038/s41598-021-03243-6 PMC8671431

[4] Lai PH, Lancet EA, Weiden MD, Webber MP, Zeig-Owens R, Hall CB et al Characteristics associated with out-of-hospital cardiac arrests and resuscitations during the novel coronavirus disease 2019 pandemic in New York City. JAMA Cardiol 2020;5:1154–63. 32558876 10.1001/jamacardio.2020.2488 PMC7305567

[5] O'Shea CJ, Thomas G, Middeldorp ME, Harper C, Elliott AD, Ray N et al Ventricular arrhythmia burden during the coronavirus disease 2019 (COVID-19) pandemic. Eur Heart J 2021;42:520–8. 33321517 10.1093/eurheartj/ehaa893 PMC7953962

[6] Leyva F, Zegard A, Okafor O, Stegemann B, Ludman P, Qiu T. Cardiac operations and interventions during the COVID-19 pandemic: a nationwide perspective. Europace 2021;23:928–36. 33778881 10.1093/europace/euab013 PMC8083650

[7] Montnach J, Baró I, Charpentier F, De Waard M, Loussouarn G. Modelling sudden cardiac death risks factors in patients with coronavirus disease of 2019: the hydroxychloroquine and azithromycin case. Europace 2021;23:1124–33. 34009333 10.1093/europace/euab043 PMC8135857

